# Safety, Efficacy, and Visual Performance of an Orthokeratology Lens with Increased Compression Factor

**DOI:** 10.3390/jcm13020587

**Published:** 2024-01-19

**Authors:** Elena Martínez-Plaza, Cecilia Zamora Castro, Ainhoa Molina-Martín, David P. Piñero

**Affiliations:** 1 Group of Optics and Visual Perception, Department of Optics, Pharmacology and Anatomy, University of Alicante, 03690 Alicante, Spain; emartinezp@ioba.med.uva.es (E.M.-P.); czc6@alu.ua.es (C.Z.C.); ainhoa.molina@ua.es (A.M.-M.); 2 University of Valladolid, 47001 Valladolid, Spain; 3 Department of Ophthalmology, Vithas Medimar International Hospital, 03016 Alicante, Spain

**Keywords:** orthokeratology, increased compression factor, safety, efficacy, visual performance

## Abstract

The aim was to evaluate the safety, efficacy, and visual performance of an orthokeratology lens with an increased compression factor (ICF) of 1.25 D in a 3-month follow-up. Thirty-six myopic patients (5 males and 31 females; 24.2 ± 5.8 years) were fitted with Alexa AR (Tiedra Farmacéutica S.L., Madrid, Spain) contact lenses (CLs) and twenty participants finished the follow-up. Visual acuity (VA), subjective refraction, primary spherical and primary coma aberrations, keratometry, central pachymetry, and ocular surface evaluation were performed at baseline and after 1 night, 1 week, 1 month, and 3 months of CL wear. The differences among visits were analyzed using a repeated-measures analysis of variance or the Friedman test. The spherical equivalent decreased (*p* ≤ 0.005), and the uncorrected VA improved (*p* < 0.001) until the first week. Corneal and ocular aberrations showed a significant increase (*p* ≤ 0.02). A significant decrease (*p* < 0.001) was found for keratometry values. No significant changes were observed in either central pachymetry or ocular surface parameters among study visits. In conclusion, an orthokeratology CL with an ICF of 1.25 D provides good safety, efficacy, and visual performance in a 3-month follow-up. Seven days of orthokeratology wear are enough to achieve the full myopic compensation, resulting in satisfactory VA.

## 1. Introduction

Orthokeratology (ortho-k) is a procedure that temporarily compensates for refractive error during the day by wearing customized rigid contact lenses (CLs) overnight [1]. The interest in the use of ortho-k has experienced a growing trend in recent years for both myopia correction and myopia control [2]. When fitting for myopia and low-to-moderate astigmatism, the cornea is reshaped by inducing a controlled central flattening along with mid-peripheral steepening [1]. Given the increasing rates of myopia progression in the coming years [3], it is expected that this refractive technique will continue to rise in popularity.

After overnight CL wear and lens removal, the ortho-k effect undergoes a regression of approximately 0.50–0.75 diopters (D) [4]. Therefore, ortho-k designs incorporate a compression factor (also widely known as the Jessen factor) that over-corrects the refractive error to compensate for this regression [5]. A conventional compression factor (CCF) of the same magnitude as the regression (0.50–0.75 D) has been traditionally used. However, numerous studies found that this overcorrection may not always be equivalent to the attempted correction [6,7,8]. Consequently, an increased compression factor (ICF) has been introduced in some ortho-k lenses.

Recent studies analyzing the effect of incorporating an ICF of 1.75 D have reported some benefits, such as an acceleration of the refractive correction [7] and better efficacy in myopia control [9]. Specifically, Wan et al. [7] found that 92% of subjects wearing a CL with an ICF of 1.75 D achieved full correction after 1 week, in comparison with 64% of CL wearers using CLs with a CCF of 0.75 D. Regarding the efficacy of myopia control, Tang et al. [9] demonstrated that an ICF of 1.75 D was 23.33% more effective in slowing axial elongation than CCF in a moderate myopia population. However, an ICF of such magnitude (1.75 D) has also been associated with an increase in higher-order aberrations, particularly spherical aberration [10]. The effect of ortho-k CLs with compression factors different from 0.75 and 1.75 D is unknown. Consequently, the aim of the present study was to evaluate the safety, efficacy, and visual performance of an ortho-k CL with an ICF of 1.25 D in a 3-month follow-up.

## 2. Materials and Methods

This prospective interventional case series study was conducted at the Optometry Clinic of the University of Alicante (Alicante, Spain). The research adhered to the principles of the Declaration of Helsinki and received ethical approval from the Ethics Committee for Medical Research of the Health Department of Alicante (General Hospital, Alicante, Spain). All enrolled participants were informed of the aim of the study, and their informed consent was obtained.

### 2.1. Sample

The present study involved 36 myopic candidates to be fitted with ortho-k CLs. Inclusion criteria were subjects with a minimum age of 18 years, a corrected distance visual acuity (VA) ≤ +0.10 logarithm of the minimum angle of resolution (logMAR), myopia of at least −0.50 D, and astigmatism below 1.50 D. Exclusion criteria were subjects with astigmatism higher than half of the sphere in subjective refraction, the presence of any ocular pathology, corneal or conjunctival staining > 1 (Oxford scale) [11], the presence of any other ocular finding that contraindicates ortho-k fitting, a previous ocular surgery, and the use of topical ocular medications.

Participants attended five follow-up visits, scheduled in the morning: the basal visit, and after one night, one week, one month, and three months of ortho-k CL wear.

### 2.2. Orthokeratology Fitting

At the first study visit, volunteers were fitted with the ortho-k CL Alexa AR (Tiedra Farmacéutica S.L, Madrid, Spain) using the contact lens-fitting set provided by the manufacturer. This lens consists of Paflufocon D material with high oxygen transmissibility (DK = 101 units). It features a four-curve design, a back-optic zone diameter of either 5.60 mm or 6.0 mm, a diameter ranging from 10.40 to 11.80 mm, and a refractive power of +1.25 D to compensate for the Jessen factor. All lenses used in the present study were standard designs, excluding lens customizations (e.g., dual axis or toric peripheries).

Once the optimal fit was achieved, subjects were instructed to wear the CLs every night and attend the Optometry Clinic after 1 night, 1 week, 1 month, and 3 months of ortho-k wear. Subjects were provided with hydrogen peroxide (VEO, Tiedra Farmaceútica S.L., Madrid, Spain) to clean and disinfect CLs, and eye drops (Aquawet, Tiedra Farmacéutica S.L., Madrid, Spain) to fill the lens before the insertion.

### 2.3. Visual Performance Evaluation

Corrected distance VA (CDVA) was recorded at the five study visits, while uncorrected distance VA (UDVA) was recorded at the four follow-up visits. Both CDVA and UDVA were measured monocularly on a logMAR scale. The safety of the procedure was considered as the percentage of eyes with a CDVA loss of two or more lines, while the safety index was calculated as the ratio of post-orthokeratology CDVA to pre-orthokeratology CDVA on a decimal scale. The efficacy index was calculated as the ratio of post-orthokeratology UDVA to pre-orthokeratology CDVA on a decimal scale.

Subjective refraction was performed at the five study visits. Spherical equivalent and cylinder were collected. Predictability was calculated considering the percentage of eyes with subjective refraction in spherical equivalent within ±0.25 D, ±0.50 D, ±0.75 D, and ±1.00 D at the 3-month visit.

Corneal, internal, and ocular aberrometry were performed at the five study visits. The Visionix 650 system (Visionix-Luneau Technology Operations, Chartres, France) was used considering an aperture of 5 mm for their calculation. Primary spherical ($Z_{0}^{4}$) and primary coma ($Z_{-1}^{3}$ and $Z_{+1}^{3}$) aberrations were recorded.

### 2.4. Corneal Morphology and Anterior Segment Integrity Evaluation

Pupil diameter (without pharmacological dilation), horizontal visible iris diameter, and intraocular pressure were measured with the Visionix 650 system (Visionix-Luneau Technology Operations, Chartres, France), at the basal visit. However, the clinical evaluation performed at the five study visits is presented below.

Simulated keratometry in the flat (SimKf) and steep (SimKs) meridians and the mean keratometry (Mean-K) were measured using the Visionix 650 system. In addition, the central corneal pachymetry was also obtained with the same device.

High-definition photographs were acquired using the Dry Eye mode of the Visionix 650 system. Bulbar and limbal hyperemia, blepharitis, and Meibomian gland dysfunction were graded using the Efron scale (range, 0–4) [12] and the tear meniscus height was quantified using the digital caliper tool [13]. In addition, the first tear film break-up (first NIBUT) and the time when half of the subareas of projection presented distortion (NIBUT50%) were also measured [12]. Finally, a slit-lamp examination (SL-8Z, Topcon, Tokyo, Japan) was performed to evaluate the corneal staining and the nasal and temporal conjunctival staining with fluorescein using the Oxford scale (range, 0–5) [11].

### 2.5. Statistical Analysis

The statistical analysis was performed using SPSS 24.0 (IBM Corp., Armonk, NY, USA). The study eye of each subject was randomly selected for statistical purposes.

The sample size was calculated to find a difference in a paired *t*-test between visits of 0.10 logMAR in VA considering a standard deviation (SD) of 0.13 (the most unfavorable SD reported by Tahhan et al. [14] in an adult sample analyzing four ortho-k CLs). A statistical significance of 0.005 (0.05/10 to control for multiple comparisons with Bonferroni correction) and a power of 90% were established. An initial sample size of 32 participants was estimated; however, a final sample size of 36 volunteers was finally recruited considering an estimated 10% dropout rate.

Descriptive data are shown as the mean and SD or median and interquartile range for numerical or ordinal variables, respectively. The normality of the numerical variables was checked using the Shapiro–Wilk test. A repeated-measures analysis of variance (ANOVA) was fitted when numerical data followed a normal distribution; otherwise, the Friedman test was used (numerical, non-normally distributed, and ordinal variables). Significant results were followed by pairwise comparisons applying the Bonferroni correction. *p*-values ≤ 0.05 were considered statistically significant.

## 3. Results

### 3.1. Study Population

A total of 36 participants (5 males and 31 females) with a mean age of 24.2 ± 5.8 years were recruited. The mean pupil diameter was 6.54 ± 0.76 mm, the mean horizontal visible iris diameter was 12.04 ± 0.51 mm, and the mean intraocular pressure was 16.77 ± 2.79 mmHg in the recruited participants at the basal visit. The 3-month follow-up was completed by 20 participants (3 males and 17 females) with a mean age of 23.9 ± 5.6 years. Their mean pupil diameter was 6.50 ± 0.81 mm, their mean horizontal visible iris diameter was 12.06 ± 0.54 mm, and their mean intraocular pressure was 16.01 ± 3.19 mmHg at the basal visit.

During the follow-up, one over-correction was detected in the first week, which was solved with a CL recalculation. Sixteen participants discontinued the CL wear: two of them (12.50%) after the 1-night evaluation, seven (43.75%) after the 1-week evaluation, and seven (43.75%) after the 1-month evaluation. The primary reasons for CL cessation were red eye and discharge (25.00%), work-related reasons (18.75%), discomfort (18.75%), handling/motivation problems (12.50%), conjunctivitis (12.50%), visual fluctuations (6.25%), and under-correction (6.25%). None of the participants experienced serious adverse events or complications.

### 3.2. Visual Performance Outcomes

**Figure 1:**
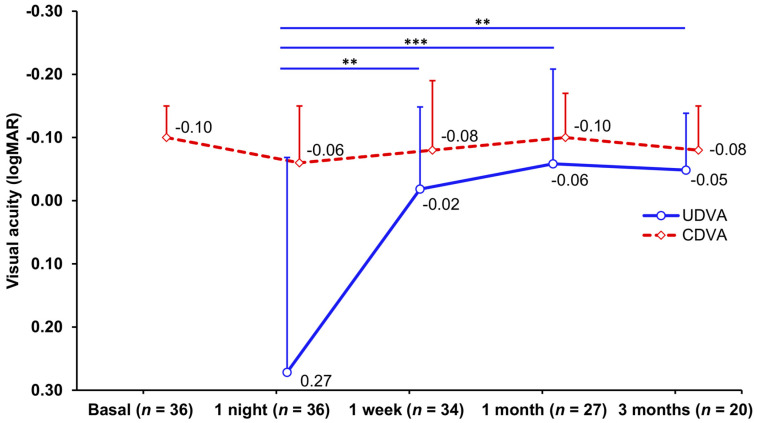
Uncorrected distance visual acuity (UDVA) and corrected distance visual acuity (CDVA) obtained at each study visit. Mean values are represented as circles and rhombuses, and standard deviation values as vertical lines. ** *p* ≤ 0.01; *** *p* ≤ 0.001.

The UDVA experienced a significant improvement (*p* < 0.001) over time. Figure 1 represents the UDVA and CDVA progression and significant differences among study visits. Two eyes (10%) lost one line, while 18 eyes (90%) neither gained nor lost one line of CDVA after 3 months of ortho-k wear. The safety index was 0.96 ± 0.13 at the 3-month visit. The efficacy index was 0.89 ± 0.17 after 3 months of ortho-k wear. 

**Figure 2:**
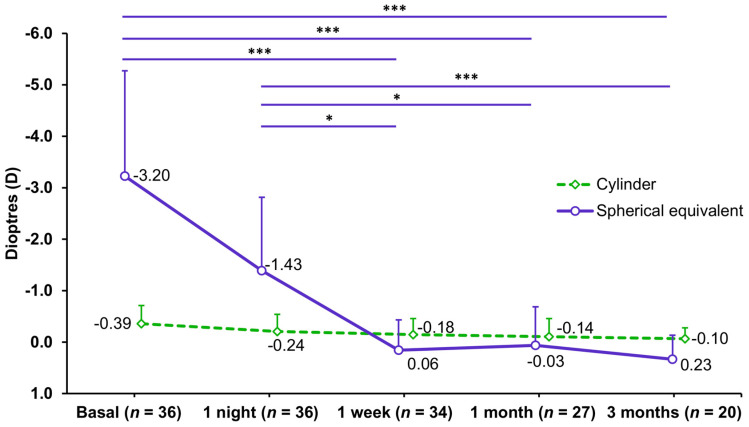
Subjective refraction obtained at each study visit. Mean values are represented as circles and rhombuses, and standard deviation values as vertical lines. * *p* ≤ 0.05; *** *p* ≤ 0.001.

The spherical equivalent showed a significant change during the ortho-k wear period (*p* ≤ 0.005). Figure 2 represents the spherical equivalent and cylinder progression and significant differences among study visits. In terms of predictability, 10 eyes (50%) had a spherical equivalent within ±0.25 D, 16 eyes (80%) were within ±0.5 D, 18 eyes (90%) were within ±0.75 D, and all eyes (100%) were within ±1.00 D at the 3-month visit.

Corneal, internal, and ocular primary spherical aberrations showed a significant increase (*p* ≤ 0.02) during the ortho-k use. Likewise, corneal, internal, and ocular primary coma aberrations experienced a significant increase (*p* ≤ 0.013) during follow-up visits. Table 1 shows the mean values and significant differences in aberrations among study visits.

### 3.3. Corneal Morphology and Anterior Segment Integrity Outcomes

A significant decrease (*p* < 0.001) was found for keratometry values (SimKs, SimKf, and Mean-K) during the ortho-k wear. In contrast, no significant changes were observed in the central pachymetry parameter over time (*p* = 0.09). Table 2 shows the mean values and significant differences in keratometry and central pachymetry among the study visits.

Significant changes were found for nasal (*p* = 0.025) and temporal (*p* = 0.016) conjunctival staining and tear meniscus height (*p* = 0.04). However, no significant changes were found for any of the evaluated ocular surface parameters among study visits. Table 3 shows descriptive data of ocular surface parameters evaluated at each study visit.

## 4. Discussion

The CL market is continuously developing novel ortho-k CL designs to compensate for ametropia and be useful for myopia control. Some ortho-k designs have recently incorporated an ICF of 1.75 D, which is suggested to reduce the time required for achieving full correction [7] but increases higher-order aberrations [10]. The Alexa AR ortho-k design incorporates an ICF of 1.25 D. We aimed to assess the clinical and visual performance of using this CL for myopia correction, finding good safety, efficacy, and predictability in a 3-month follow-up.

The UDVA reached the target VA (≤0.00 logMAR) after 1 week and remained stable for up to the 3 months of ortho-k wear. The CDVA showed a similar magnitude during all study visits. In addition, the safety index showed that the pre- and post-orthokeratology CDVAs were almost equal, while the efficacy index revealed that the pre-orthokeratology CDVA was slightly better than the post-orthokeratology UDVA. This aligns with recent studies assessing adults [15,16,17] and children wearing CCF and ICF CLs [18,19], which have obtained similar VA outcomes to ours, substantiating the established idea regarding the good efficacy of the ortho-k procedure [20].

The subjective refraction, in terms of spherical equivalent, decreased by a magnitude of approximately half of the refractive error after 1 night. Full compensation was achieved after 1 week and was maintained for up to the 3 months of Alexa AR wear. Previous studies assessing ICF (1.75 D) CLs found a similar trend, observing the full compensation after 1 week, in contrast to CCF (0.75 D) CLs, which required a slightly longer period [7,17]. Despite the rapid correction, our results did not show a relevant overcorrection using ICF CLs of 1.25 D for 1 and 3 months (−0.03 D in a range from −0.75 to +0.50 D, and 0.23 D in a range from −0.75 to +1.00 D, respectively). However, CLs with an ICF of 1.75 D frequently show an overcorrection around 0.25–0.50 D after 1 month of ortho-k wear [7,18,21]; whereas the CCF (0.75 D) CLs hardly show overcorrection [7,18,21]. Particularly, Lau et al. [18] reported, after 1 month, an overcorrection of +0.44 D (range from −1.13 to +1.50 D) with an ICF of 1.75 D and 0.00 D (range from −1.00 to +1.00 D) with a CCF of 0.75 D. Then, although the ranges among different compression factors appear to be similar, the mean values indicate that the higher the compression factor, the more rapid and greater the myopia correction.

The change in refractive correction is caused by the corneal reshaping induced during the ortho-k wear, flattening the central cornea and steepening the mid-peripheral area [1]. The decrease in keratometry values observed reflects the central flattening. This effect was maintained up to 1 week of ortho-k wear, aligned in time with the refractive compensation. The central pachymetry values decreased from 1 week, whose magnitude was similar to that reported in a meta-analysis, which showed a mean reduction of 6 µm after 1 week and 1 month of ortho-k wear compared to baseline [22]. However, overall central pachymetry did not show significant changes during the ortho-k period. The repeatability of central pachymetry measurements is an important issue [23], whose consistency has been demonstrated using the Visionix 120 system [24,25]. Additional potential reasons may include differences in the magnitude corrected or sample variability, or discrepancies in the applied pressure compared to other CLs. The latest hypothesis agrees with the lack of central sagittal height change observed after using Alexa AR [26].

The corneal reshaping induced by ortho-k wear has been reported to alter both corneal and ocular spherical aberration [10]. These findings are in line with the increase in the magnitude of spherical aberration found in the present study. However, there is controversy about whether the compression factor influences the magnitude of spherical aberration. Lau et al. [10] found a greater increase using ICF (1.75 D) CLs in comparison with CCF (0.75 D) ones in children, while He et al. [17] did not in adults. Regarding coma aberration, its increase in ortho-k wearers has been associated with CL decentration [27]. Given that CL decentration can be found even after a successful CL-fitting process [28], the increase in coma aberration found in our sample could be the consequence of slight lens decentrations. Thus, the aberrations found in the present study after the use of CLs featuring an ICF of 1.25 D appear to be similar to those reported for other ortho-k CL models. It is remarkable that the degradation in image quality is not high enough to prevent subjects from achieving a satisfactory visual acuity.

In the current study, ocular surface parameters (such as corneal and conjunctival integrity, hyperemia, or NIBUT, among others) were not altered during the follow-up. There is considerable literature reporting the safety of the procedure in short and long terms [29]. On the other hand, in the present study, sixteen CL discontinuations were registered during the follow-up. The main reasons for CL cessation were red eye and discharge, discomfort, and work-related reasons. None of the causes that motivated the CL discontinuations can be considered to be serious adverse events according to the classification by Morgan et al. [30].

The primary limitation of the current study is the absence of a control group. However, the aim was to assess the safety, efficacy, and visual performance of the ortho-k lens Alexa AR in a 3-month follow-up. In addition, variations in daytime among visits might have affected the outcomes; nevertheless, this impact was minimized by scheduling all study visits in the morning. Future research could evaluate the performance of Alexa AR in longer periods and conduct comparative analyses with other ortho-k CLs available in the market. Furthermore, it could also be interesting to collect patient-reported outcomes as well as to study the effect of an ortho-k CL with an ICF of 1.25 D on myopia control. 

## 5. Conclusions

In summary, correcting myopia with ortho-k CLs with an ICF of 1.25 D (Alexa AR) provides good safety, efficacy, and visual performance in a 3-month follow-up. Seven days of ortho-k wear are enough to achieve the full refractive compensation, resulting in subsequent satisfactory visual acuity. Future works are encouraged to corroborate our results in a longer follow-up and analyze the behavior of CLs with an ICF of 1.25 D in a myopic child population. 

## Figures and Tables

**Table 1:** Mean values of corneal, internal, and ocular aberrations and significant differences among study visits.

Parameters	Basal (*n* = 36)	1 Night (*n* = 36)	1 Week (*n* = 34)	1 Month (*n* = 27)	3 Months (*n* = 20)
Corneal $Z_{0}^{4}$ (µm)	0.09 ± 0.04 ^a,b,c,d^	0.25 ± 0.14 ^a,e,f^	0.37 ± 0.22 ^b,e^	0.33 ± 0.20 ^c,f^	0.35 ± 0.24 ^d^
Internal $Z_{0}^{4}$ (µm)	−0.07 ± 0.06	−0.04 ± 0.09	−0.03 ± 0.09	−0.02 ± 0.09	−0.02 ± 0.09
Ocular $Z_{0}^{4}$ (µm)	0.02 ± 0.06 ^a,b,c,d^	0.21 ± 0.15 ^a,e,f^	0.33 ± 0.25 ^b,e^	0.30 ± 0.21 ^c,f^	0.33 ± 0.30 ^d^
Corneal $Z_{+1}^{3}$ (µm)	0.11 (0.02/0.22) ^b,d^	0.20 (0.03/0.73)	0.20 (0.03/0.98) ^b^	0.23 (0.02/1.42)	0.27 (0.07/1.06) ^d^
Internal $Z_{+1}^{3}$ (µm)	0.13 (0.03/0.20) ^d^	0.11 (0.02/0.39)	0.14 (0.03/0.71)	0.13 (0.02/0.51)	0.17 (0.04/0.53) ^d^
Ocular $Z_{+1}^{3}$ (µm)	0.08 (0.01/0.22) ^b,c,d^	0.20 (0.05/0.52)	0.32 (0.06/1.13) ^b^	0.27 (0.08/1.10) ^c^	0.39 (0.02/0.16) ^d^

Data are presented as mean ± standard deviation for normally distributed variables and as median (interquartile range) for non-normally distributed variables. $Z_{0}^{4}$: primary spherical aberration; $Z_{+1}^{3}$: primary coma aberration. ^a^ Statistically significant difference between the basal and 1-night visits (*p* < 0.01). ^b^ Statistically significant difference between the basal and 1-week visits (*p* < 0.01). ^c^ Statistically significant difference between the basal and 1-month visits (*p* < 0.01). ^d^ Statistically significant difference between the basal and 3-month visits (*p* < 0.01). ^e^ Statistically significant difference between the 1-night and 1-week visits (*p* < 0.01). ^f^ Statistically significant difference between the 1-night and 1-month visits (*p* ≤ 0.05).

**Table 2:** Mean values and significant differences of keratometry and central pachymetry among study visits.

Parameters	Basal (*n* = 36)	1 Night (*n* = 36)	1 Week (*n* = 34)	1 Month (*n* = 27)	3 Months (*n* = 20)
SimKf (D)	43.56 ± 1.74 ^a,b,c,d^	42.92 ± 1.69 ^a,e,f,g^	42.05 ± 1.95 ^b,e^	41.83 ± 1.70 ^c,f^	41.86 ± 1.69 ^d,g^
SimKs (D)	44.44 ± 1.74 ^a,b,c,d^	43.86 ± 1.68 ^a,e,f,g^	42.98 ± 1.89 ^b,e^	42.74 ± 1.78 ^c,f^	43.07 ± 1.67 ^d,g^
Mean-K (D)	44.00 ± 1.72 ^a,b,c,d^	43.39 ± 1.67 ^a,e,f,g^	42.46 ± 1.90 ^b,e^	42.29 ± 1.71 ^c,f^	42.47 ± 1.64 ^d,g^
Central pachymetry (µm)	544.75 ± 37.70	552.12 ± 45.22	538.39 ± 40.14	542.56 ± 40.22	534.35 ± 33.01

Data are presented as mean ± standard deviation. D: diopters; Mean-K: mean keratometry; SimKs: simulated keratometry in the steep meridian; SimKf: simulated keratometry in the flat meridian. ^a^ Statistically significant difference between the basal and 1-night visits (*p* < 0.05). ^b^ Statistically significant difference between the basal and 1-week visits (*p* ≤ 0.001). ^c^ Statistically significant difference between the basal and 1-month visits (*p* ≤ 0.001). ^d^ Statistically significant difference between the basal and 3-month visits (*p* < 0.001). ^e^ Statistically significant difference between the 1-night and 1-week visits (*p* ≤ 0.01). ^f^ Statistically significant difference between the 1-night and 1-month visits (*p* < 0.01). ^g^ Statistically significant difference between the 1-night and 3-month visits (*p* < 0.01).

**Table 3:** Descriptive results of the ocular surface parameters at each study visit.

Parameters	Basal (*n* = 36)	1 Night (*n* = 36)	1 Week (*n* = 34)	1 Month (*n* = 27)	3 Months (*n* = 20)
Conjunctival hyperemia	1.00 (0/3.00)	1.00 (0/2.00)	1.00 (0/2.00)	1.00 (0/3.00)	1.50 (0/2.00)
Limbal hyperemia	1.00 (0/2.00)	1.00 (0/2.00)	1.00 (0/2.00)	1.00 (0/2.00)	1.00 (0/2.00)
Blepharitis	0 (0/1.00)	0 (0/1.00)	0 (0/1.00)	0 (0/1.00)	0.50 (0/1.00)
MGD	0 (0/1.00)	0 (0/1.00)	0 (0/2.00)	0 (0/1.00)	0 (0/0)
First NIBUT	4.05 (2.80/13.80)	2.90 (2.80/13.60)	3.60 (2.80/13.60)	3.00 (2.80/13.60)	2.90 (2.20/17.00)
NIBUT 50%	7.60 (3.50/15.50)	7.05 (1.70/13.60)	6.90 (3.20/15.20)	7.70 (3.20/14.70)	7.25 (3.20/13.60)
TMH	0.17 ± 0.05	0.19 ± 0.06	0.19 ± 0.05	0.17 ± 0.05	0.16 ± 0.04
Corneal staining	0 (0/1.00)	0 (0/1.00)	0 (0/4.00)	0 (0/2.00)	0 (0/2.00)
Nasal conjunctival staining	0 (0/1.00)	0 (0/2.00)	0 (0/2.00)	0 (0/3.00)	0 (0/1.00)
Temporal conjunctival staining	0 (0/0)	0 (0/1.00)	0 (0/2.00)	0 (0/1.00)	0 (0/1.00)

Data are presented as mean ± standard deviation for numerical, normally distributed variables and as median (interquartile range) for numerical, non-normally distributed, or ordinal variables. MGD: Meibomian gland degeneration; NIBUT: non-invasive tear break-up time; TMH: tear meniscus height.

## Data Availability

Data are contained within the article.
